# Acquired Immune Deficiency Syndrome Status in Islamic Countries

**DOI:** 10.5812/ijhrba.6578

**Published:** 2012-11-26

**Authors:** Roya Alavi-Naini

**Affiliations:** 1 Infectious Disease and Tropical Medicine Research Center, Zahedan University of Medical Sciences, Zahedan, IR Iran

**Keywords:** Acquired Immune Deficiency Syndrome, Islam, Human Immunodeficiency Virus

*Editorial*

Acquired Immune Deficiency Syndrome (AIDS) is a social-health problem. Injection drug use, sharing needles and sexual contacts are considered as the main ways of Human Immunodeficiency Virus (HIV) transmission from person to person in Central Asian countries as well as Iran (1). In recent years, the number of people who have died from AIDS is declining while; the number of HIV carriers is also rising. Therefore, the risk of the disease transmission by apparently healthy individuals is alarming. Reduction of risky sexual behaviors has been urged in Islamic canonical principles (Shariat) which has led to a decrease in the incidence of HIV infections in Muslims. On the other hands, prohibition of alcohol use in Islamic codes has resulted in reduction of abnormal behaviors and ultimately decreases the rate of sexually transmitted diseases such as AIDS. Besides, circumcision of Muslim boys as well as cleaning and washing of body after intercourse (ablution) can be enumerated as one of the influential factors in reduction of this disease which in turn can dramatically reduce the other infections incidence. However, some beliefs such as failing to encourage condom use and polygamy could lead to increase the risk of some sexually transmitted infections. In general, since the detection of HIV, African countries have been reported as the major zone infected with the HIV. Based on the surveys of the World Health Organization, Sub-Saharan Africa experienced noticeable decrease in AIDS infection incidence (2). In conducted surveys, six out of seven published articles with the subject of the relationship between religious beliefs and AIDS indicate that AIDS epidemic among the Muslims of Sub-Saharan Africa is less than non-Muslims citizens (3). with reference to World Health Organization reports, Central Asia and Eastern Europe are facing with the alarming increase of known cases of HIV, in such a way that the spread of HIV in some countries in the Middle East and North Africa, including Egypt, Sudan and Tunisia, has been growing and warns among some Islamic countries like an epidemic. It should be noted that the registered cases of HIV infection in many of these countries are much lower than the estimated cases declared by the World Health Organization (4). Obviously, Iran is not an exception. Despite low prevalence of AIDS in different regions of Iran, it is expected that, in case of improper control and lack of adequate training and education, we will confront an increase in its prevalence around the country. Fortunately, continuous efforts in recent years on this basis have been very valuable in Iran, which is known as the best country in the field of AIDS control in the Middle East, but still there is a long way to achieve the desired result.
